# COVID-19 Vaccine Acceptance and Its Determinants among Myanmar Migrant Workers in Southern Thailand

**DOI:** 10.3390/ijerph192013420

**Published:** 2022-10-17

**Authors:** Kanit Hnuploy, Kittipong Sornlorm, Than Kyaw Soe, Patthanasak Khammaneechan, Navarat Rakchart, Wajinee Jongjit, Suttakarn Supaviboolas, Nirachon Chutipattana

**Affiliations:** 1 Faculty of Science and Technology, Suratthani Rajabhat University, Suratthani 84100, Thailand; 2 Faculty of Public Health, Khon Kaen University, Khon Kean 40002, Thailand; 3 UNICEF Myanmar, Nay Pyi Taw 15015, Myanmar; 4 Excellent Centre for Dengue and Community Public Health (E.C. for DACH), School of Public Health, Walailak University, Nakhon Si Thammarat 80161, Thailand; 5 School of Nursing, Walailak University, Nakhon Si Thammarat 80161, Thailand; 6 Department of Public Health Strategy Development, Nakhon Si Thammarat Provincial Public Health Office, Nakhon Si Thammarat 80000, Thailand; 7 Southern Border Regional Center for Primary Health Care Development, Nakhon Si Thammarat 80000, Thailand

**Keywords:** COVID-19 vaccine, vaccine acceptance, Myanmar, migrant worker, Thailand

## Abstract

Success in eradicating COVID-19 will rely on the rate of vaccination adoption worldwide. Vaccine acceptance among vulnerable groups is critical for preventing the spread of COVID-19 and decreasing unnecessary deaths. The purpose of this study was to report on the willingness to obtain COVID-19 immunization and the factors related to its acceptance among Myanmar migrant workers in southern Thailand. This cross-sectional study consisted of 301 samples collected between October and November 2021 and analyzed using multiple logistic regression. Thirty-nine percent of workers intended to receive the COVID-19 vaccine within a year. The following factors were associated with obtaining the COVID-19 vaccine: a high level of perception of COVID-19 (AOR = 5.43), income less than or equal to 10,000 baht/month (AOR = 6.98), financial status at a sufficient level (AOR = 7.79), wearing a face mask in the previous month almost all the time (AOR =4.26), maintaining 1–2 m of distance from anyone in the last month (AOR =2.51), and measuring temperature in the previous month (AOR = 5.24). High reluctance to accept the COVID-19 vaccine among Myanmar migrant workers can influence efforts to eliminate COVID-19. Collaboration with all stakeholders is critical to helping Myanmar workers understand COVID-19, social measures, and preventive beliefs to increase vaccine uptake.

## 1. Introduction

Coronavirus disease 2019 (COVID-19) is a respiratory infectious disease caused by the virus known as severe acute respiratory syndrome coronavirus-2 (SARS-CoV-2) [1]. The first identified case was in Wuhan, China, in December 2019. SARS-CoV-2 can disseminate through direct (droplet and human-to-human transmission) and indirect contact (contaminated objects and airborne contagion) [2]. The World Health Organization (WHO) declared the COVID-19 pandemic on 11 March 2020, and this pandemic has had a broad and terrible impact on global health and the economy [1,3]. There had been 175,541,600 confirmed cases of COVID-19 as of 14 June 2021, with 3,798,361 deaths worldwide.

Vaccines serve a crucial role in preventing deaths and hospitalizations caused by infectious diseases and help to control disease transmission; hence, their influence on infection and severe illness is enormous [4]. However, widespread vaccine hesitancy can limit vaccine uptake and effectiveness as vaccine hesitancy encompasses a range of vaccination-related attitudes and beliefs that result in delayed or outright refusal of immunization [5]. It is vital to look into vaccine acceptance or reluctance in order to inform the national response to COVID-19.

Vaccines are one of the most dependable and cost-effective public health interventions ever implemented and save millions of lives annually [6]. With the development of SARS-CoV-2 vaccinations, there is growing hope that the pandemic may end through herd immunity. The predicted SARS-CoV-2 herd immunity threshold is between 50% and 67% [7]. A systematic review study showed that vaccine acceptance of the COVID-19 vaccination ranged from 19% to 89% in the surveyed migrant population [8]. Therefore, it is worth investigating the variables that motivate migrants to receive the vaccination. Furthermore, emerging research from numerous countries explores the factors that influence COVID-19 vaccination acceptance [9,10,11]. Most studies have focused on the general population [9,10,11], while migrant workers, who are recognized as a vulnerable group, have received less attention [12].

Thailand purchased COVID-19 vaccinations directly from the manufacturers [13]. The Thai government did not participate in the COVID-19 Vaccines Global Access (COVAX) program [14,15], although the migrant-rich Caribbean area did [16]. The Caribbean region’s average percentage of fully immunized people is 29–68%. Even though Thailand received immunizations after that zone, vaccine coverage in Thailand rapidly increased, reaching 80% (73% fully vaccinated and 7% partially vaccinated) by April 2022 [17].

Thailand offers vaccinations to migrant workers through various channels, including government, civil society networks, entrepreneurs, and self-payment [18]. The government provides free immunizations to all country residents, and people can pay for optional vaccines [17]. However, the number of doses supplied to foreign nationals accounted for 3% of total national doses administered on 30 September 2021 [19]. Myanmar nationals received the most partial immunization with 481,546 doses. As per Thai policy, migrant workers and their family members will have full access to COVID-19 vaccines [17]. It is unknown whether they will get the vaccination when it becomes available.

Myanmar is Thailand’s most engaged nation [20], and travel between Thailand and Myanmar, people’s home countries, caused the second wave of the Thai pandemic in December 2020 [21]. Due to a lack of vaccines at that time in Thailand, both nationals and migrant workers received very few doses. Infection among workers in the seafood market spurred local transmission to over 30 areas. Coronavirus virus 2019 infected approximately half of the 4000 workers from Myanmar [21].

Health and well-being are possible consequences of COVID-19, as are anxiety, depression, lower wages, and job loss [22]. A high prevalence of illness in Myanmar may increase the load of healthcare services, contributing to the country’s growing healthcare expenditure [23,24]. In addition, Myanmar provides an essential workforce in the tourism, construction, and service sectors, which impacts the country’s economic growth [25].

Migrant workers’ risk of COVID-19 infection is likely to be enhanced due to their frequent and intimate contact with the public [12]. Acceptance of vaccines is critical, and multiple factors influence vaccination uptake. Socio-demographics, preventive measures, knowledge, and health beliefs affect the desire to receive COVID-19 vaccinations [26]. People who earn lower incomes, for example, are more willing to accept the vaccine than those who earn higher incomes [27]. Workers who report higher mask wearing are more likely to obtain a COVID-19 vaccine [28], and higher knowledge levels indicate higher vaccination intention [29]. One of the most commonly used theories in the study of health and illness behavior is the health belief model (HBM), which comprises five key components: perceived susceptibility, perceived severity, perceived benefits, perceived barriers, and cues to action. This model has been demonstrated to be a crucial predictor of the intention to receive COVID-19 vaccines [26]. For instance, people who believe the vaccine decreases the chance of infection and makes them feel less worried will have a definite intention to receive it [30].

There has been little research on vaccine acceptance and its determinants among migrant workers, with a few studies emerging in China, Bangladesh, and Qatar [8,12,31]. In China, most migrants (89.1%) accept the COVID-19 vaccine. Belief in the necessity, safety, and efficiency of the COVID-19 vaccine is strongly related to vaccine acceptance [12]. Overall vaccination reluctance in Bangladesh is 25%, with significant variation by the host country. The strongest predictor of COVID-19 vaccination reluctance is threat perception [31]. In Qatar, 20.2% of respondents said they would not accept the vaccination, while 19.8% said they were undecided about accepting the potential COVID-19 vaccine. The primary concerns raised were the safety of the COVID-19 vaccination and its long-term adverse effects [8].

The government and policymakers need to implement effective vaccination strategies. They must address the prevalence and factors related to vaccine acceptance to remove all barriers to COVID-19 vaccine uptake. There has been no previous research on the acceptance of the COVID-19 vaccine among Myanmar migrant workers. As a result, this study aimed to examine acceptance of the COVID-19 vaccine and identify the factors that influence it.

## 2. Materials and Methods

### 2.1. Research Design

This research is a cross-sectional study conducted between October and November 2021. The setting of this study was southern Thailand. The population was composed of Myanmar migrant workers. The inclusion criteria were: being a Myanmar national, having a working permit certificate, working in South Thailand for at least six months, being between 18 and 60 years old, being able to communicate with researchers verbally, and agreeing to participate in the study with written informed consent. Serious illness-related exclusions applied to participants.

The sample size was estimated using Hsieh’s formula for logistic regression analysis [32]. Using a multistage random sampling procedure, we obtained 301 estimated sample sizes. The random sampling procedure was as follows. Step 1: the researchers examined 14 provinces in the southern area, seven in the lower southern zone, and seven in the upper southern zone by choosing one province from each site using simple random sampling; the researchers selected Songkhla and Surat Thani at random. Step 2: based on simple random sampling, an area contained two districts. Step 3: Probability proportional to size (PPS) sampling was conducted based on the population of those districts. Step 4: the researchers chose each district’s sample using systematic random sampling.

### 2.2. Measures

A questionnaire in Supplementary Materials was developed based on the research questions and relevant literature [27,28,30,33,34]. The questionnaire in Supplementary Materials consisted of five parts.

Part I included thirteen items on socio-demographics, such as gender, age, and highest level of education. Respondents also answered questions about their health and lifestyle, including influenza vaccination, belief that the government and the public health system could deal with the COVID-19 pandemic, and receiving information.

Part II included ten items on preventive measures. The researchers asked respondents to rate the frequency with which they followed Thailand’s social actions in the previous month. To assess social measures, respondents chose one of four responses: ‘never’, ‘sometimes’, ‘almost every time’, and ‘every time’ [28]. Respondents also provided information about Thailand’s application installation and behavior related to the risk of COVID-19. There were two response options: ‘yes’ and ‘no’.

Part III included twelve items on COVID-19 knowledge, such as the cause, transmission, symptoms, prevention, and treatment. Respondents chose one correct response from a list of multiple choices. Those who correctly answered the question earned one point, while those who replied incorrectly received zero points. The level of knowledge was categorized, using Bloom’s cut-off point, as low (0–7.1 points), moderate (7.2–9.5 points), and high (7.6–12 points) [35].

Part IV had nineteen items on COVID-19-preventative health beliefs. The HBM constructs section included five domains: susceptibility perception, infection severity perceptions, obstacles and benefits of COVID-19 vaccination beliefs, and cues to action. The researcher asked the respondents to rate each statement with 3 points for ‘agree’, 2 points for ‘unsure’, and 1 point for ‘disagree’. The perception ratings were divided into low (19–31 points), moderate (32–43 points), and high (44–57 points) [36].

Part V included one item on the intention to receive the COVID-19 vaccine. The researcher asked respondents to indicate whether they would be willing to receive the COVID-19 vaccine within a year by selecting ‘yes’ or ‘no’ [33].

The three experts assessed the items and provided comments on the content validity of the Thai questionnaire in Supplementary Materials with an item-objective congruence index of 0.66-1. The researchers made revisions to increase its validity. A total of 30 respondents took part in the pilot study using Burmese questions under survey settings similar to those used in the main study. Cronbach’s alpha coefficient was 0.89 for health belief, and the Kuder–Richardson 20 reliability was 0.76 for knowledge. The surveys were available in two versions: Thai and Burmese. A bilingual individual translated from Thai to English, and a medical doctor from Myanmar translated from English to Burmese.

The researcher addressed an official letter to the chief of the provincial public health office requesting authorization to gather data on Myanmar migrant workers. Three study assistants completed standardized data collection skills training. The research assistants then interviewed Myanmar workers in their habitat.

The study procedure was well-designed and controlled; there was no missing data in this study. Use simple words, for example, and keep the inquiries short and to the point. The researchers supervised research assistants. The assistant researchers questioned each respondent face-to-face and checked all items to avoid data loss.

### 2.3. Statistical Analysis

A simple logistic regression was used for bivariate analysis to identify the association of each independent variable with acceptance of the COVID-19 vaccine. There were two categories, yes and no. Independent variables with a *p*-value less than 0.25 were analyzed for multivariable analysis using multiple logistic regression to identify factors associated with accepting the COVID-19 vaccine [37]. The backwards elimination method was used as the model fitting strategy. The magnitude of association was presented as an adjusted odds ratio (Adj. OR) with a 95% confidence interval (CI), and the statistically significant level was a *p*-value < 0.05. The Hosmer–Lemeshow goodness of fit test, with an alpha of 0.05, revealed that the final model fits the data. The result was interpreted as having no association if AOR was 1, an association if AOR was more than 1, and a protective effect if AOR was less than 1.

## 3. Results

### 3.1. Socio-Demographics of Participants

Table 1 shows the socio-demographics of the sample. Most respondents were male (55.48%) and adults (43.19% aged 31–40). More than half of them had completed secondary school (57.81%). Almost all were Buddhists (95.68%), employees (94.35%), and not vaccinated against influenza in the past year (97.34%). Nearly one-fourth of them had a medical condition (23.26%). Most had an income of THB 10,001–20,000 (37.54%). Approximately two-thirds had insufficient but debt-free financial status (65.45%). They rarely believed that the government could deal with the COVID-19 pandemic (43.85%). Almost half did not believe the government would be able to deal with the COVID-19 pandemic (43.85%), and nearly one-third did not think the public health system would be able to deal with COVID-19 (37.87%). Nearly four in five respondents (79.73%) reported receiving health-related information on COVID-19, with non-governmental organization employees providing most of the content (53.82%).

### 3.2. COVID-19 Prevention Social Measures

Table 2 depicts the previous month’s compliance with COVID-19 preventative social measures. Almost half of them wore a face mask (41.86%), washed their hands with alcohol or soap (35.55%), and checked their body temperature (33.88%) consistently. Half of them were occasionally 1–2 m from other people (42.19%). Almost none of them downloaded and installed the MorChana application to track their presence (86.71%). Most never scanned QR codes with the ThaiChana application for check-in at various sites to assist healthcare professionals in promptly investigating the disease (35.88%). Only 3.99% were exposed to any known cases, 2.66% were tested for COVID-19, and 96.35% had never visited any risky locations.

### 3.3. COVID-19-Preventive Health Beliefs and Knowledge

Table 3 depicts the understanding of COVID-19 and the perception of disease prevention. Most respondents had minimal knowledge (84.38%), while just a few had a high degree of expertise (8.31%). The majority of them (69.10%) regarded COVID-19 prevention as moderate. One in every four people (26.58%) reported having a high level of perception.

### 3.4. COVID-19 Vaccine Uptake Intentions

Table 4 indicates the prevalence of respondents who wanted to receive the COVID-19 vaccine within a year. A total of 60.80% did not plan to receive the COVID-19 vaccination, whereas 39.20% intended to receive it.

### 3.5. Factors Associated with Willingness to Receive COVID-19

Table 5 displays the predictors associated with the intent to receive the vaccine. Respondents with a monthly income of THB 10,001–20,000, less than or equal to THB 10,000, and a good financial situation were more likely to be willing to receive the COVID-19 vaccine. Respondents who perceived a high level of preventive COVID-19 measures wore a face mask almost always or always, never or sometimes remained 1–2 m away from anyone, and almost always or always measured their body temperature were more likely to accept the vaccine.

The results revealed that respondents with good financial status had a 7.79 times greater intention to receive the COVID-19 vaccine than people with poor economic conditions. Those who had an average monthly income of less than or equal to THB 10,000 were 6.98 times more likely to intend to receive the COVID-19 vaccine than people who had an average monthly income of more than THB 20,000. Respondents with a high perception were 5.43 times more likely to receive the COVID-19 vaccine than workers with a low perception. The participants who always followed social measures, such as temperature measurements and wearing a mask outside, were more likely to receive the COVID-19 vaccine than those who did so occasionally or never in the past month, at 5.24 times and 4.26 times, respectively. People who never or occasionally remained 1–2 m away from people outside their family were 2.51 times more likely to intend to receive COVID-19 vaccines than workers who did so often or every time in the past month.

## 4. Discussion

Our survey showed that 39.2% of Myanmar migrant workers were willing to receive the COVID-19 vaccine. It is probable that the respondents had insufficient knowledge and were not overly concerned about preventing COVID-19. Only 26% kept a distance of 1–2 m when meeting people outside their families, and approximately 36% always wore masks when leaving the house and washed their hands. In contrast, a study conducted in China found that 89.1% of migrants accepted the COVID-19 vaccination, and over 90% of migrants believed that the COVID-19 vaccine was important. That study reported high acceptance of other personal protective measures, such as mask use, hand cleaning, and social distancing [12]. Hence, health authorities should move quickly to provide immunizations to Myanmar workers and encourage the uptake of the COVID-19 vaccine because persons infected with COVID-19 will incur enormous healthcare expenses for the government [38].

The most significant factor influencing COVID-19 vaccine intent is the financial situation. Financially secure workers were more likely to intend to be vaccinated. According to a Nigerian study, people with the highest wealth index were ten times more likely to want to obtain the COVID-19 vaccine than those with the lowest wealth index [39]. Individuals with sufficient financial conditions may be able to purchase the COVID-19 vaccination, but those in a poor financial situation will not have enough money to do so.

Moreover, the more low-income workers there were, the more likely they were to choose to vaccinate. It is conceivable that the former group may be aware of the advantages of vaccines, which enable them to work and earn an income. In a study in Russia, people who earned low incomes were more willing to receive the vaccine than those who earned high incomes [27]. In contrast to research conducted in Bangladesh, more people with higher incomes planned to receive the vaccination than those with lower incomes because the former group perceived the disease to be more severe and riskier and had more knowledge about vaccinations [40]. However, most Myanmar workers have a low income and insufficient financial status, so policymakers must support free vaccination services to increase acceptance rates.

Furthermore, workers were more likely to be vaccinated if they positively perceived vaccination. It is feasible that highly perceptive workers obtained NGO information and had confidence in vaccinations. Previous research indicated that the crucial causes of vaccination hesitancy among migrant workers were a lack of vaccine information and trust, such as belief in vaccine importance, safety, and efficacy [12]. Similarly, a Lebanese study found that obtaining reliable and adequate vaccination information and recommendations from health authorities and health facilities was positively related to vaccine acceptance [41]. In the present study, most workers perceived COVID-19 prevention at a moderate level. Health authorities can use this information to implement suitable programs, such as conducting migrant volunteer training on COVID-19 understanding to provide accurate vaccination information to Myanmar workers. A previous study indicated that migrants face vaccination access challenges, primarily due to language and communication problems [42]. Hence, the participation of migrant volunteers will considerably diminish the language barrier.

Additionally, workers who consistently took their temperatures and wore masks outside were more inclined to accept the vaccine. Workers who comply with social norms are probably interested in self-prevention. According to a study conducted in China, wearing a mask and washing hands are positively related to the acceptance of COVID-19 vaccines [28]. People who wear masks perceive that if they are infected, they will become seriously ill; therefore, they wear masks and receive vaccines [43]. To enhance vaccine acceptability, health workers must encourage Myanmar workers to wear masks and suggest temperature measures before going out, as well as provide information on the severity of the disease and the advantages of vaccines.

Interestingly, workers who could not keep their distance from other people had a higher chance of deciding to be vaccinated. Most workers work in jobs that require them to interact with many people, such as in the tourism, construction, and service sectors. Working in enclosed spaces with others makes them believe they are at high risk of catching the virus. Similarly, a Chinese factory workers’ study revealed that workers in crowded places intended to receive the COVID-19 vaccine [28]. In contrast to a Saskatchewan survey in which those who maintained social isolation and had limited or no interaction with people outside their homes were concerned about spreading the virus and were more likely to engage in vaccine acceptance [43]. Differences in sample characteristics might explain this mismatch. Elderly people in Saskatchewan, who have little engagement with others, desired vaccination; they perceived that they were at greater risk of infection and mortality than people of other ages. While the participants in our study were concerned about the spread of disease due to contact with many people, they wanted to be vaccinated.

In addition, Myanmar migrant workers in Thailand were the most affected by COVID-19 infections in adults and children as of September 30, 2021 [19]. The Ministry of Public Health Immunization Center administered COVID-19 vaccines to migrants in Thailand. Myanmar nationals had the highest rate of partial vaccination, accounting for 55% of all Myanmar workers. Most migrant workers live in large groups in specially built accommodations, including communal facilities and community places for sharing meals and activities [41,42]. Social distancing is difficult to practice, and the attributes of such an environment may not be sufficient, including the inability to isolate themselves in the case of sickness that causes COVID-19 to spread quickly. Therefore, health authorities should engage with migrant workers’ employers to ensure safe working environments and dining areas to reduce the risk of infection. During COVID-19, health practitioners may design patterns or activities to generate learning processes for health literacy, such as creating learning spaces and expressing themselves to promote healthy behavior.

### Limitations

Despite the results stated above, this study has some limitations. This cross-sectional study presented a correlation explanation but did not investigate the causal relationship. It would be an interesting issue for future research to identify the causal relationship using different study designs, such as a cohort study. The findings are useful as a reference point. However, they do not apply to other Myanmar migrant workers studying in Thailand or other countries. Future research should examine different settings and have a larger sample size. Furthermore, because acceptance of the COVID-19 vaccination may shift, researchers may focus on long-term COVID-19 vaccination among Myanmar migrant workers.

## 5. Conclusions

Unfortunately, only 39.20% of Myanmar migrant workers intended to accept the COVID-19 vaccines within a year. Good financial status, low income, a high perception of preventive measures against COVID-19, constant masking and temperature measurements, and inability to maintain distance influenced COVID-19 vaccine acceptance. The health authority should encourage healthcare practitioners to engage with migrant employers, local governments, and migrant volunteers to help Myanmar workers understand COVID-19 and social measures, including perceptions about preventative illness and the COVID-19 vaccine, to enhance vaccination rates.

## Figures and Tables

**Table 1:** Socio-demographic factors of Myanmar migrant workers (*n* = 301).

Factors	Number	Percentage
Gender		
Male	167	55.48
Female	134	44.52
Age (years)		
Less than or equal to 30	95	31.56
31–40	130	43.19
41–50	61	20.27
More than or equal to 51	15	4.98
Mean (SD)	33.84 (8.92)	
Median (Min–Max)	34 (18–58)	
Highest level of education		
Uneducated	18	5.98
Primary school	85	28.24
Secondary school	174	57.81
Bachelor’s degree	19	6.31
Higher than bachelor’s degree	5	1.66
Religion		
Buddhism	288	95.68
Christianity	10	3.32
Islam	3	1.00
Occupation		
Employee	284	94.35
Unemployed	4	1.33
Agriculturist	4	1.33
Merchant	2	0.66
Others	7	2.33
Medical condition		
No	231	76.74
Yes	70	23.26
Income (baht/month)		
Less than or equal to 10,000	87	28.90
10,001–20,000	113	37.54
20,001–30,000	34	11.30
30,001–40,000	38	12.62
40,001–50,000	27	8.97
Above 50,000	2	0.67
Financial status		
Insufficient and indebted	65	21.59
Insufficient but debt-free	197	65.45
Sufficient without savings	12	3.99
Sufficient with savings	27	8.97
Vaccinated against influenza in the past year		
Yes	8	2.66
No	293	97.34
Believe that the government can deal with the COVID-19 pandemic		
Strongly believe	80	26.58
Rarely believe	132	43.85
Do not believe	89	29.57
Believe the public health system can cope with COVID-19		
Strongly believe	99	32.89
Rarely believe	114	37.87
Do not believe	88	29.24
Provided with health information concerning COVID-19		
No	61	20.27
Yes	240	79.73
Channel that provides such information		
Officer of non-governmental organization (NGOs)	162	53.82
Radio/Television	110	36.54
Internet/Facebook/Line	69	22.92
Friends	66	21.93
Village health volunteer	47	15.61
Public health officer	10	3.32
Foreign volunteer	9	2.99
Public relations sign	6	1.99

**Table 2:** Compliance with COVID-19 prevention social measures during the previous month (n = 301).

Items	Number	Percentage
Wore a face mask		
Never	44	14.62
Sometimes	38	12.62
Almost every time	93	30.90
Every time	126	41.86
Washed your hands with alcohol or soap		
Never	31	10.30
Sometimes	99	32.89
Almost every time	64	21.26
Every time	107	35.55
1–2 m distance from anyone		
Never	43	14.29
Sometimes	127	42.19
Almost every time	51	16.94
Every time	80	26.58
Measured your body temperature		
Never	67	22.26
Sometimes	88	29.24
Almost every time	44	14.62
Every time	102	33.88
Scanned QR codes through the ThaiChana application		
Never	108	35.88
Sometimes	62	20.60
Almost every time	95	31.56
Every time	36	11.96
Downloaded and installed the MorChana application to track your location		
Yes	40	13.29
No	261	86.71
Exposed to any confirmed cases		
No	289	96.01
Yes	12	3.99
Tested for COVID-19 infection		
Yes	8	2.66
No	4	1.33
Travelled to any risky areas		
No	290	96.35
Yes	11	3.65
Tested for COVID-19 infection		
Yes	3	1.00
No	8	2.65

**Table 3:** Number, percentage, and 95% confidence interval of COVID-19 knowledge and perception (*n* = 301).

Factors	Number	Percentage	95% CI
Knowledge			
Low (0–7.1)	254	84.38	79.81–88.07
Moderate (7.2–9.5)	22	7.31	4.85–10.86
High (9.6–12)	25	8.31	5.66–12.02
Perception of prevention			
Low (19–31)	13	4.32	2.51–7.30
Moderate (32–43)	208	69.10	63.63–74.08
High (44–57)	80	26.58	21.87–31.87

**Table 4:** Number, percent, and 95% confidence interval of Myanmar migrant workers who wanted to receive the COVID-19 vaccine within a year (*n* = 301).

Intention to Receive the COVID-19 Vaccine	Number	Percentage	95% CI
No	183	60.80	55.14–66.18
Yes	118	39.20	33.82–44.86

**Table 5:** Unadjusted and adjusted logistic regression analyses of factors associated with the acceptance of the COVID-19 vaccine (*n* = 301).

Factors	Number	% Intend to Receive	Crude OR	Adj. OR	95% CI	*p*-Value
Income (baht/month)						
Above 20,000	101	13.86	1	1	1	<0.001
10,001–20,000	113	40.71	4.26	2.71	1.25–5.87	
Less than or equal to 10,000	87	66.67	12.42	6.98	2.99–16.29	
Financial status						<0.001
Insufficient	262	32.82	1	1	1	
Sufficient	39	82.05	9.35	7.79	2.86–21.21	
Level of perception of COVID-19						<0.001
Low–Moderate	221	27.60	1	1	1	
High	80	71.25	6.50	5.43	2.63–11.22	
Wore a face mask in the past month						0.003
Never–Sometimes	82	23.17	1	1	1	
Almost every time–Every time	219	45.21	2.73	4.26	1.65–10.96	
1–2 m distance from anyone in the past month						0.034
Almost every time–Every time	131	32.82	1	1	1	
Never–Sometimes	170	44.12	1.61	2.51	1.07–5.88	
Measured body temperature in the past month						<0.001
Never–Sometimes	155	28.39	1	1	1	
Almost every time–Every time	146	50.68	2.59	5.24	2.41–11.38	

## Data Availability

The full dataset presented in this study is available on request from the corresponding author.

## Supplementary Materials

The following supporting information can be downloaded at: https://www.mdpi.com/article/10.3390/ijerph192013420/s1, Questionnaire: COVID-19 Vaccine Acceptance among Myanmar Migrant Workers in Southern Thailand.
