# Supplementary material for: COVID-19 Vaccine Acceptance and Its Determinants among Myanmar Migrant Workers in Southern Thailand
Source: Int J Environ Res Public Health. 2022 Oct 17;19(20):13420. doi: 10.3390/ijerph192013420 (PMC9602811; doi:10.3390/ijerph192013420)
Supplement: Supplementary file 1 [file ijerph-19-13420-s001.zip › ijerph-1958041-supplementary.pdf]

Part I: Information concerning personal characteristics, economy, and social
Instruction: Please mark "X" in "☐" matching to your characteristics or fill the relevant information in the blanks

Item	Inquiry
A1	You are currently …….. years old (Monthly fraction exceeding 6 months is deemed 1 year)
A2	Born gender<br>☐ 1. Male ☐ 2. Female
A3	Highest education<br>☐ 1. Uneducated ☐ 2. Primary school ☐ 3. High School<br>☐ 4. Bachelor's Degree ☐ 5. Higher than Bachelor's Degree<br>☐ 6. Other (Please specify) …………………………………..
A4	Religion<br>☐ 1. Buddhism ☐ 2. Islam ☐ 3. Christianity<br>☐ 4. Other (Please specify) ……………………………………
A5	Occupation<br>☐ 1. Unemployed/jobless ☐ 2. Merchant ☐ 3. Employee<br>☐ 4. Agriculturist ☐ 5. Self-employed<br>☐ 6. Public official/Government employee/Government officer<br>☐ 7. Other (Please specify) ……………………………………
A6	Do you have any medical conditions?<br>☐ 1. Null<br>☐ 2. Yes.
A7	How much do you earn monthly (Baht/month)?<br>☐ 1. Less than or equal 10,000 ☐ 2. 10,001 – 20,000 ☐ 3. 20,001 – 30,000<br>☐ 4. 30,001 – 40,000 ☐ 5. 40,001 – 50,000 ☐ 6. Above 50,000
A8	How is your financial status?<br>☐ 1. Insufficient and indebted ☐ 2. Insufficient but debt-free<br>☐ 3. Sufficient without saving ☐ 4. Sufficient with saving
A9	During the last one-year period, have you had an influenza vaccination?<br>☐ 1. Yes ☐ 2. No
A10	How much do you believe that the Government could deal with the COVID-19 pandemic?<br>☐ 1. Very believe ☐ 2. Rarely believe ☐ 3. Do not believe
A11	How much do you believe the public health system could cope with COVID-19?<br>☐ 1. Very believe ☐ 2. Rarely believe ☐ 3. Do not believe
A12	Are you provided with the health information concerning COVID-19?<br>☐ 1. No (if the answer is "No", please skip to B1)<br>☐ 2. Yes (please be directed to A13)
A13	Which channel does provide you such information? (Multiple choices are allowed)<br>☐ 1. Public health officer ☐ 2. Village public health volunteer<br>☐ 3. Foreign volunteer ☐ 4. Officer of non-governmental organization (NGO)<br>☐ 5. Friend ☐ 6. Public relations sign<br>☐ 7. Radio/Television ☐ 8. Internet/Facebook/Line<br>☐ 9. Other (Please specify) ……………………………

Part II: Information concerning the COVID-19 preventive measure

Instruction: Please mark "X" in "☐" matching to your behaviors within the last 1 month.

Item	Inquiry
B1	During the last one-month period, how often you have worn a face mask while staying outside the house/meeting with anyone other than your family members?<br>☐ 1. Never ☐ 2. Sometimes<br>☐ 3. Almost every time ☐ 4. Every time
B2	During the last one-month period, how often you have washed your hands with alcohol or soap while staying outside the house/meeting with anyone other than your family members?<br>☐ 1. Never ☐ 2. Sometimes<br>☐ 3. Almost every time ☐ 4. Every time
B3	During the last one-month period, how often do you have 1-2 meters distanced from anyone other than your family members?<br>☐ 1. Never ☐ 2. Sometimes<br>☐ 3. Almost every time ☐ 4. Every time
B4	During the last one-month period, how often you have measured your body temperature while being at places?<br>☐ 1. Never ☐ 2. Sometimes<br>☐ 3. Almost every time ☐ 4. Every time
B5	During the last one-month period, how often you have scanned the QR codes through the ThaiChana application before entering and exiting public venues such as shops?<br>☐ 1. Never ☐ 2. Sometimes<br>☐ 3. Almost every time ☐ 4. Every time
B6	Have you downloaded and installed the MorChana application for monitoring your being at places (timeline)?<br>☐ 1. Yes. ☐ 2. No.
B7	During the last one-month period, have you been exposed to any confirmed cases?<br>☐ 1. No. (If the answer is "No", please skip to B9)<br>☐ 2. Yes. Be directed to B8
B8	Have you been tested for the COVID-19 infection?<br>☐ 1. Yes. ☐ 2. No.
B9	During the last one-month period, have you traveled to any risky areas (provinces marked with dark red, red, and orange)?<br>☐ 1. No. (If the answer is "No", please skip to K1)<br>☐ 2. Yes. Please be directed to B10.
B10	Have you been tested for the COVID-19 infection?<br>☐ 1. Yes. ☐ 2. No.

Figure S1

Depicts provincial risk areas (to be used in conjunction with item B9)

Zoning of COVID-19 risk areas by province		
**Color zone**	**Number of provinces**	**List of provinces**
Dark red	29	Angthong, Bangkok, Chachoengsao, Chonburi, Kan Chanaburi, Lopburi, Nakhon Nayok, Nakhon Pathom, Nakhon Ratchasima, Narathiwat, Nonthaburi, Pathum Thani, Pattani, Phetchabun, Phetchaburi, Phra Nakhon Si Ayutthaya, Prachinburi, Prachuap Khiri Khan, Ratchaburi, Rayong, Samut Prakan, Samut Sakhon, Samut Songkhram, Saraburi, Singburi, Songkhla, Suphanburi, Tak, and Yala
Red	37	Amnat Charoen, Buriram, Chai Nat, Chaiyaphum, Chanthaburi, Chiang Mai, Chiang Rai, Chumphon, Kalasin, Kamphaeng Phet, Khon Kaen, Lampang, Lamphun, Loei, Maha Sarakham, Nakhon Sawan, Nakhon Si Thammarat, Nong Bua Lamphu, Nong Khai, Phatthalung, Phichit, Phitsanulok, Ranong, Roi Et, Sa Kaeo, Sakon Nakhon, Satun, Si Sa Ket, Sukhothai, Surin, Trang, Trat, Ubon Ratchathani, Udon Thani, Uthai Thani, Uttaradit and Yasothon
Orange	11	Bueng Kan, Krabi, Mae Hong Son, Mukdahan, Nakhon Phanom, Nan, Phangnga, Phayao, Phrae, Phuket, and Surat Thani

Part III: Information concerning COVID-19 knowledge

Instruction: Please mark "✓" in "☐" matching to your single best answer

Item	Inquiry
K1	Which pathogen originates COVID-19?<br>☐ 1. Bacteria<br>☐ 2. Virus<br>☐ 3. Fungus
K2	Which is the best career for the spread of COVID-19?<br>☐ 1. Aerosol of nasal mucus and saliva<br>☐ 2. Dry air<br>☐ 3. The surface of material or object
K3	Is the COVID-19 spread to humans by the same means as influenza?<br>☐ 1. Yes ☐ 2. No ☐ 3. Unsure
K4	What are the symptoms often found in a COVID-19-infected person?<br>☐ 1. Fever, cough, exhaustion<br>☐ 2. Diarrhea<br>☐ 3. Skin rash
K5	How many days of quarantine when the COVID-19 infection is suspected?<br>☐ 1. 7 days<br>☐ 2. 14 days<br>☐ 3. 20 days
K6	What should risky persons with the first COVID-19-positive result perform?<br>☐ 1. Being quarantined<br>☐ 2. Quarantine is unnecessary<br>☐ 3. Either quarantine or not depending on availability
K7	How long do you wash your hands for the COVID-19 prevention?<br>☐ 1. 5 seconds ☐ 2. 10 seconds ☐ 3. 20 seconds
K8	Notwithstanding washing hands with water and soap, what is concentration of alcohol should be used for disinfection?<br>☐ 1. 10-20%<br>☐ 2. 30-50%<br>☐ 3. 60-70%
K9	Could an asymptomatic person spread the COVID-19?<br>☐ 1. Yes ☐ 2. No ☐ 3. Unsure
K10	Could the person having a 2-dose COVID-19 vaccination be infected or ill?<br>☐ 1. Both are possible<br>☐ 2. Both are impossible due to immunity<br>☐ 3. Unsure
K11	Could a person having infected COVID-19 be re-infected?<br>☐ 1. Yes ☐ 2. No ☐ 3. Unsure
K12	Is that true that, currently, there is no efficient treatment for COVID-19 but the initial treatment leads to the patient's rapid rehabilitation?<br>☐ 1. Yes ☐ 2. No ☐ 3. Unsure

Part IV: Information concerning the COVID-19 perception

Instruction: Please mark “X” in “☐” best matching to your situations. The answers consist of 3 choices: disagreed, unsure, and agreed

Item	Inquiry	Agreed	Unsure	Disagreed
	**Perception on Risk of COVID-19**			
P1	You are at high risk to be infected with COVID-19.			
P2	Without being vaccinated, you will be risky of the COVID-19 infection.			
P3	You are concerned about being infected with COVID-19.			
	**Perception on the COVID-19 Severity**			
P4	You will get serious illness upon the COVID-19 infection.			
P5	COVID-19 can lead to mortality?			
P6	COVID-19 is more severe than influenza.			
P7	If the COVID-19 virus translocates to the lungs and prompt treatment is not performed, this could lead to mortality.			
	**Perception on Benefits of the COVID-19 Treatment and Prevention**			
P8	The COVID-19 preventive vaccination causes body immunity.			
P9	The COVID-19 preventive vaccination mitigates the severity of symptoms upon infection.			
P10	The COVID-19 preventive vaccination mitigates your mortality rate due to COVID-19.			
P11	The COVID-19 preventive vaccination enables you to seek jobs, merchandise, and travel to any place.			
	**Perception on Obstacles against the COVID-19 Preventive Vaccination**			
P12	You are scared by the side effects of the COVID-19 vaccine.			
P13	You believe that the domestic COVID-19 vaccines are capable of mitigating the rate of severe illness or mortality therefrom.			
P14	You do not know how to reserve the COVID-19 vaccines.			
P15	COVID-19 vaccines reservation can be done difficultly.			
	**Reasons Persuading into Practices during the COVID-19 Pandemic**			
P16	You will receive the COVID-19 preventive vaccine if you are suggested by medical and public health personnel.			
P17	You will receive the COVID-19 preventive vaccine if the government sector provides you with the choices of vaccines.			
P18	You will receive the COVID-19 preventive vaccine			

Item	Inquiry	Agreed	Unsure	Disagreed
	if you will be paid by the government upon vaccine allergy.			
P19	You decide to receive the vaccine upon receiving information from various types of media.			

Part V: Information concerning determination to accept vaccination

1. Do you intend to receive the COVID-19 preventive vaccine within a year?

☐ 1. Yes ☐ 2. No
